# Alveolar bone regeneration using sustainable eggshell-derived nano-hydroxyapatite and zinc oxide nanoparticles: an in vivo experimental study

**DOI:** 10.1038/s41598-025-22899-y

**Published:** 2025-11-18

**Authors:** Eman M. Salem, Rehab M. El-Sharkawy, Wafaa Yahia Alghonemy, Omnia M. Abdelfatah, Rania A. Hanafy

**Affiliations:** 1 Oral Biology Department, Faculty of Dentistry, Pharos University in Alexandria, Sidi Gaber, P.O. Box 37, Alexandria, Egypt; 2 Chemistry Department, Faculty of Dentistry, Pharos University in Alexandria, Sidi Gaber, P.O. Box 37, Alexandria, Egypt; 3 Basic Dental Sciences Department, Faculty of Dentistry, Zarqa University, Zarqa, Jordan; 4 Department of Oral Biology, Faculty of Dentistry, Tanta University, Tanta, Egypt; 5 Dental Materials Department, Faculty of Dentistry, Pharos University in Alexandria, Sidi Gaber, P.O. Box 37, Alexandria, Egypt

**Keywords:** Nanomaterials, Bone healing, Tooth extraction, NHAP, ZnO NPs, Developmental biology, Medical research

## Abstract

The present study focused on developing nanocrystalline hydroxyapatite (NHAP) from waste eggshells and assessed its role in bone regeneration after tooth extraction, with and without the addition of zinc oxide nanoparticles (ZnO NPs). Sixty adult male albino rats were used. The lower first molar was extracted, and the rats were divided into four groups (*n* = 15): Group I, control without treatment; Group II, bone socket loaded with ZnO NPs; Group III, bone socket loaded with NHAP; and Group IV, bone socket treated with both ZnO NPs and NHAP. Animal scarification was performed 4 weeks after extraction, and the results were assessed using histological, histomorphometric, and scanning electron microscopic assessment of bone. Also, energy dispersive X-ray analysis (EDXA) was performed for all animal groups and subsequently evaluated statistically. After 4 weeks of extraction, Group I exhibited complete healing with remnants of immature bone, while in Groups II and III, mature compact bone filled most of the socket, and cancellous bone occupied small areas. Group IV exhibited complete healing of the extraction socket, which was completely closed by mature bone. The combined use of NHAP derived sustainably from eggshells and laboratory-synthesized ZnO NPs proposes a potential, biocompatible, and economical approach for improving alveolar socket healing. These nanomaterials enhance osteoinduction and antibacterial properties and exemplify a novel application of locally obtained waste and cost-effective synthesis methods, rendering them highly appropriate for clinical utilization in resource-constrained environments, as in developing countries. Thus, dentists can access and benefit from state-of-the-art nanotechnological solutions without financial impediments, as in alveolar ridge preservation and rapid healing after extraction for future implant placement. Treatment modalities with ZnO NPs and NHAP administered separately could accelerate bone socket healing. However, combining ZnO NPs and NHAP resulted in a more rapid improvement of the histological and statistical results. This might be due to the enhancement of the early release of growth factors that increase angiogenesis and regeneration in healing sockets. However, additional studies are needed to evaluate this combination.

## Introduction

Nanomaterials with bone-inducing properties have been developed to be among the best graft materials for preventing bone loss after tooth extraction. Nano-hydroxyapatite (NHAP) is one of these materials. It has the benefit of being a bioresorbable and osteoconductive material that works as a scaffold for new bone growth by controlling bone cell activity^1,2^. Inspired by the biological features of natural bone, many studies have reported various synthesis routes to obtain nano-hydroxyapatite with controlled shape and size to produce NHAP with properties like biological apatite. There is also a strong connection between the production method of the NHAP and its clinical application and properties. Solid-state and wet chemical methods have been the most common for synthesizing NHAP. The first method involves milling and calcination to obtain Nano powders from hydroxyapatite (HAP) precursors (biochemical reagents or biowastes). The chemical method is the most popular since it is more amenable to tuning the size and morphology of NHAPs^3,4^.

Zinc oxide nanoparticles (ZnO NPs) exhibit desirable characteristics, including low cytotoxicity, antimicrobial, and antioxidant activities. They have recently been researched for use in many dental applications, as they can enhance the cellular differentiation of various tissues. They are also easy and inexpensive to prepare^5^. Regarding bone formation, ZnO NPs enhance collagen synthesis, control alkaline phosphatase activity, and play essential roles in bone mineralization and osteocalcin expression in the osteoblast cells. Scientists have recently discovered that Zn and ZnO NPs are necessary for healthy bones. Many studies have shown that they are critical in preserving the function of the bones and joints and supporting bone density by helping the body metabolize calcium and magnesium. They are also vital in osteogenesis^6,7^. In addition to the above-reported orthopaedic advantages, ZnO NPs have great bactericidal activity. Thus, they are added to dental materials, disinfectants, and pharmaceuticals^8^.

Although NHAP has been widely studied and applied in bone regeneration, including alveolar ridge preservation^9,10^, its bioactivity enhancement by incorporating zinc oxide nanoparticles (ZnO NPs) remains largely unexplored. While previous studies have investigated NHAP derived from eggshells, the combined in vivo application of NHAP and ZnO NPs, particularly for accelerating post-extraction socket healing, has not, to our knowledge, been previously reported. Therefore, the present study aims to investigate, in vivo, the regenerative potential of NHAP and ZnO NPs, individually and in combination, to evaluate their synergistic effect on enhancing post-extraction socket healing in an experimental albino rat model. Accordingly, this study proposes a novel, sustainable, and cost-effective nanomaterial-based strategy for bone regeneration in dental applications. This approach harnesses the advantages of nanotechnology while maintaining economic feasibility and promoting clinical applicability.

The PICO question was as follows: The population was male albino rats. After tooth extraction, the intervention groups were ZnO NPs, NHAP, and ZnO NPs + NHAP. The control group was left for spontaneous healing. The outcome was to evaluate the bone formation after tooth extraction in all groups.

## Materials and methods

All animal experimental procedures were approved by the protocols established by the Research Ethics Committee of the Faculty of Dentistry, Pharos University, under ethical code PUA02202312313166. All methods were carried out per the relevant guidelines and regulations, including the ARRIVE guidelines and institutional animal welfare standards.

### Sample size calculation

The minimum sample size was calculated based on a previous study on the effect of boron and fish oil on the healing of extraction sockets in rats^11^. A standardized effect size in OTM (primary outcome) of 0.8140 was detected with an adoption power of 80% (β = 0.20) at a significance level of 5% (acceptable α error = 0.05). The negligible necessary sample size was 15 per Group (Total sample size 15 × 4 = 60 specimens) (time points = 1)^12^. Thus, 60 male albino rats were considered the total number of animals. Any specimens lost from the research sample due to processing errors shall be replaced to preserve the sample size.

### Group assignment and animal preparation

Sixty adult male Sprague-Dawley albino rats aged 8–10 weeks (200–250 g) with regular dentition were acquired from the animal house of Pharos University, Egypt, two weeks before teeth extraction. The selection of rats for the experiment was conducted randomly. All selected rats underwent a thorough general health and oral examination. Animals showing any signs of systemic illness, malformations, dental abnormalities, or oral pathology were excluded to ensure homogeneity of the study sample and to eliminate confounding factors. The animals received food and water ad libitum throughout the experimental period and were housed under standard laboratory conditions with controlled temperature (22 ± 2 °C) and a 12-hour light/dark cycle, in accordance with institutional animal care guidelines and the ARRIVE recommendations^13–15^.

### Tooth extraction procedures

Animals from all groups were given antibiotics orally: spiramycin 7 mg/kg (Pharaoina Pharmaceuticals, Egypt) and metronidazole 12 mg/kg (Sanofi Aventis, Egypt) three days before teeth extraction for experimental infection control^16^. Before anaesthesia, rats were fasted for 2–4 h, with free access to water up to the time of the procedure.

For bilateral mandibular first molar tooth extraction, an intramuscular injection of 0. 4 ml/Kg of atropine sulfate was accomplished under general anesthesia, followed by an infusion of a mixture of xylazine 2% (Adwia, 10th of Ramadan City, Egypt) and ketamine hydrochloride 10% (ketamine alfasan10%, Woerden, The Netherlands) at a dose of 0.2 and 0.5 ml/Kg body weight, respectively^17,18^.

Animals from all groups were subjected to bilateral mandibular first molar tooth extraction according to the protocol of Moghadam et al., 2024^19^. First, an iodine swab was applied to the surgical site. Then, each tooth was luxated using surgical elevators by tipping it slowly in the buccal direction. In the lingual direction, for 1 s each, this was repeated 10 times until the tooth was loosened. After this step, the tooth was easily extracted with the lower remaining root extraction forceps. Any specimen with an intraoperative root fracture would have been excluded and replaced.

After tooth extraction procedures, a total of 60 rats were categorized randomly into 4 groups (*n* = 15 rats per Group):

*In Group I (+ ve control*), the extraction sockets were left without application of any material.

*In Group II* (*ZnO NPs*), the extraction sockets were loaded with ZnO NPs.

*In Group III (NHAP)*, the extraction sockets were loaded with NHAP.

*In Group IV (ZnO NPs + NHAP)*, the extraction sockets were loaded with a mixture of ZnO NPs and NHAP.

After materials were administered in groups II, III, and IV, the socket was filled with the formulated material, sufficient to fill the socket, and flushed with the alveolar bone margins without overpacking^20,21^, the extraction sockets were sutured with 4 − 0 black silk sutures^22^. Then, all animal groups received Ampicillin 25 mg/kg (Misr Co., Egypt) body weight every eight hours for five days and Cataflam (IM) (Novartis, Egypt) every eight hours for two days. Throughout the first week, every animal was monitored daily to see whether any indications of infection or inflammation were observed.

### Materials preparation

The materials used in the synthesis of NHAP include calcium carbonate (CaCO_3_) derived from eggshells, which was sourced from residential areas, as well as disodium hydrogen phosphate (Na_2_HPO_4_, FW 292.24 g/mol, and assay > 99%) and ethylene diamine tetra acetic acid (EDTA, C_10_H_16_N_2_O_8_, FW 141.96 g/mol, and assay > 99%), both obtained from Merck, Germany. A Millipore Simplicity unit was used to purify deionized water to a resistivity of Z18.2 MΩ, which was uniformly applied in all experimental procedures. Zinc acetate dihydrate (Zn (CH_3_COO)_2_·2H_2_O, FW 219.56 g/mol, with a purity exceeding 98.5%) was sourced from BDH in the UK. Sodium hydroxide (NaOH, FW 40.0 g/mol, with a purity greater than 99%) was sourced from Merck Company in Germany. Ethanol (C_2_H_5_OH, FW 46.07 g/mol and a purity level of 99.8%) was procured from BDH Chemicals in England. Urea (CO(NH_2_)_2_), obtained from the Indian Oxford Company, has a molecular weight of 60.0 g/mol and a purity above 99%. This substance served as fuel for the synthesis of ZnO NPs. All the substances employed, including reagents, solvents, and chemicals, were of analytical grade and were utilized directly without undergoing additional purification.

#### Synthesis of ZnO NPs

Following the method outlined by Mahmoud et al.,^23^  a straightforward combustion approach was employed to produce ZnO NPs. In this process, 100 mL of NaOH solution (0.5 M) was gradually introduced to 500 mL of zinc acetate dihydrate (Zn (CH_3_COO)_2_ · 2H_2_O) solution (0.05 M). The solution was heated at 60 °C until the reaction was fully completed. During a stirring period of 90 min, the temperature increased to 80 °C, resulting in a white precipitate. This precipitate, identified as zinc hydroxide (Zn (OH)_2_), was collected by decantation. The following steps consist of washing the resulting precipitate three times using deionized water, then drying it in an oven overnight at a temperature of 70 °C. To proceed with the combustion synthesis, the dried zinc hydroxide was mixed with stoichiometric amounts of urea and a small volume of deionized water to form a uniform reaction medium (appearing as a viscous phase). This intermediate mixture was dried at 70 °C for 24 h. Finally, the dried product was calcined at 500 °C for 6 h to obtain ZnO nanoparticles in fine powder form, which were later used in the experimental procedures.

#### Microwave-assisted synthesis of NHAP

Following the methodology presented by Ali et al.^24^, NHAP was synthesized from waste derived from eggshells. In this process, eggshells are assembled and placed in hot water to remove the internal layer and surface dirt. To eliminate moisture, eggshells were dried in a microwave oven for 24 h at a temperature of 110 °C. Once the eggshells dried, they were ground into powder, treated with sodium hypochlorite, and stirred for 12 h to eliminate organic matter. The outcome undergoes a systematic rinsing with deionized water and is subsequently dried in an oven at approximately 110 °C for 5 h. The following step includes the dissolution of 0.1 M EDTA in deionized water. Gradually, calcium carbonate sourced from eggshells is then incorporated into the EDTA solution, resulting in the formation of the Ca-EDTA complex. Afterward, Na_2_HPO_4_ with a concentration of 0.06 M is introduced drop-wise to the Ca-EDTA composite solution. The stirring lasts 30 min until a transparent and clear reaction mixture is achieved. A 0.1 M NaOH solution is utilized to adjust the pH of the resulting mixture to 13. The prepared mixture is then exposed to microwave irradiation in a microwave oven operating at 2.45 GHz and 900 W for 15 min. After irradiation, the resulting white precipitate is rinsed with deionized water and then placed in an oven at 110 °C for 5 h to dry.

#### Combination of NHAP and ZnO NPs

In a 4:1 ratio, 3.2 g of NHAP was mixed with 0.8 g of ZnO nanopowder and combined with distilled water to produce a new 4 g slurry formulation that features a moderate concentration of ZnO NPs (20%)^25,26^. Subsequently, an adequate amount of the thickening agent (polyethylene glycol) liquid was introduced to ensure the formulation reached the desired consistency, allowing the operator to handle the formulation effectively within the socket. The newly designed formulation, consisting of NHAP and ZnO NPs, was soaked in 70% ethanol for 15 min for disinfection. It was then rinsed twice with deionized water and allowed to dry. Afterward, the formulation was placed in a clean bottle and stored in the refrigerator.

### Instrumentation for characterization of the synthesized ZnO NPs and NHAP

During this study, various characterization procedures were applied to investigate the structural features of the synthesized ZnO NPs and NHAP samples. Table 1 provides comprehensive details regarding these instruments.

**Table 1:** Instruments used and their specifications.

Instrument name	Model	Data	Conditions
Fourier-transform infrared spectrophotometer (FT-IR)	A BRUKER VERTEX 70	FT-IR spectrum	400–4000 cm^− 1^
X-ray diffraction (XRD)	Shimadzu Lab x 6100, Kyoto, Japan	XRD spectrum	The XRD generator works at 40 kV, 30 mA, λ = 1 Å, using target Cu-Kα with secondary monochromatic X-ray, 2θ from 10° to 80°, recording steps of the diffraction data of 0.02 °, at a time of 0.6 s, at room temperature (25 °C)
Scanning electron microscope (SEM)	JSM-lT200, JEOL Ltd Sputtering coating (JEOL-JFC-1100E)	SEM images	Imaging mode
High-resolution transmission electron microscope (HR-TEM)	JEOL- JSM-1400 plus	TEM image	Imaging mode
Energy dispersive X-ray (EDX)	JSM-lT200, JEOL Ltd	EDX spectrum	Acceleration voltage 20.00 kV, WD 10.00 mm, live time 30.00, high vacuum mode

### Animal euthanization

Following a four-week healing period post-extraction of the mandibular first molars, all animals were humanely euthanized via intramuscular administration of an overdose of xylazine hydrochloride (30 mg/kg; Adwia, 10^th^ of Ramadan City, Egypt) and ketamine hydrochloride (70 mg/kg; Ketamine Alfasan 10%, Woerden, The Netherlands)^27^. Subsequently, the mandibles were carefully dissected, and the left halves corresponding to the first molar regions were excised. The harvested segments were processed for light microscopic evaluation, scanning electron microscopy (SEM), ultrastructural analysis, and energy-dispersive X-ray spectroscopy (EDX).

### Histological evaluation

After mandible dissection, the left half of each mandible was prepared for light microscopic (LM) assessments to investigate the histological alterations in tissue structure between the groups. Samples were fixed in 10% formalin, decalcified with 10% Ethylenediaminetetraacetic Acid (EDTA) solution (pH 7.0–7.4) at room temperature, and had regular solution changes twice weekly; decalcification required nearly four weeks. After that, the tissue is thoroughly washed in running water, then processed routinely and placed into paraffin blocks following the accepted protocol^28^. After sectioning the samples (5 μm sections), staining with hematoxylin and eosin (H&E), for both quantitative and qualitative assessments, and Masson’s Trichrome was used for qualitative evaluation of collagen deposition and bone matrix maturation^29^. Histological analysis under LM was performed with a digital camera (Leica ICC50 HD), and pictures of characteristic areas were taken and labelled.

### LM histomorphometric analysis

The morphometric examination was performed on the captured images from LM analysis to evaluate new bone formation (percentage) in the four groups^30^. H&E-stained sections were imaged at X100 magnification and analyzed in ImageJ: the new bone area was divided by total socket area to yield percent new bone. Images were analyzed using ImageJ software, version 1.46 (National Institutes of Health, Bethesda, MD, USA).

### Scanning electron microscopy (SEM) evaluation

After carefully removing the soft tissues, all groups’ bone surfaces corresponding to the first molar of the right mandibular half were observed using an SEM Model Quanta 250 FEG (Field Emission Gun). The samples were then carefully dried and gold-plated using a JFC-1100E-IEOL ion sputtering evaporator. They were then fixed on an SEM (JEOL-JSM-5200LV, Tokyo, Japan)^31^.

###  Energy dispersive X-ray analysis (EDX)

The molar region from the right half of each jaw was sectioned and prepared for EDX to evaluate the percentages of calcium and phosphorus in the alveolar bone among the four groups^32^.

### Statistical analysis

The LM histomorphometric and EDX analysis data were entered into the computer, and IBM SPSS version 20.0 (Armonk, NY: IBM Corp.) was used for the analysis. ANOVA was used to compare the overall differences between the control and the other three groups, followed by a post hoc test (Tukey). The significance of the acquired results was determined at the 5% level. Statistical probability will predict the likelihood of the different outcomes of the three experimental groups^33^.

## Results

### Clinical observations

During the experiment, the general behavior of the rats in the five groups was observed. They had normal behavior and no apparent change in their body weight. All animal treatment groups healed without significant complications and were sacrificed as scheduled. There was no dropout of rats during the experiment.

### Structural characterization of the synthesized ZnO NPs and NHAP

#### FT-IR spectroscopy

**Fig. 1:**
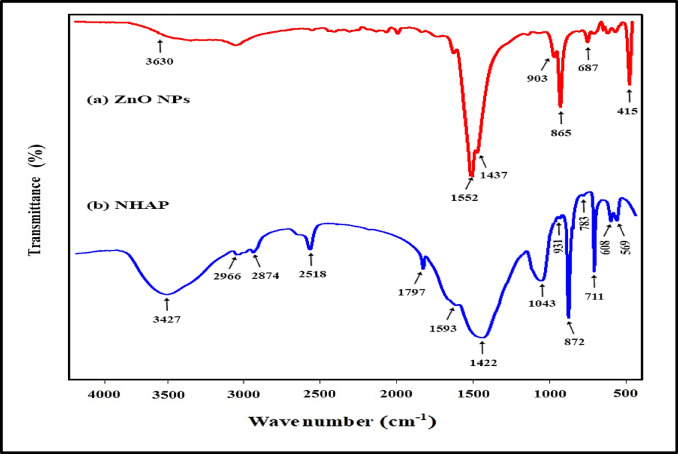
FT-IR spectra, (**a**) ZnO NPs displaying six significant peaks at the following wavenumbers: 1437, 1552, 865, 687, 903, and 415 cm^− 1^. The existence of a peak at 415 cm^− 1^ is linked to the Zn–O stretching vibration, which validates the creation of the final product. The absorption of moisture led to the appearance of two bands at approximately 1437 and 3630 cm^− 1^, which are attributed to the stretching and bending vibrations of the H–O–H molecule, respectively. Moreover, the notable peak at 1552 cm^− 1^ is due to the strong metal-oxygen bond found in ZnO. Other less intense bands were recorded at 687, 865, and 903 cm^− 1^, which relate to the ZnO nanoparticles^23,32^. (**b**) NHAPs are marked by diverse vibrational modes of the phosphate and hydroxyl phosphate groups. The peaks identified at 569, 608, 711, 783, 872, 931, and 1043 cm^− 1^ correspond to the vibrations of the tetrahedral phosphate group in NHAP, serving as further evidence for the formation of hydroxyapatite^33^. The peak located at 3427 cm^− 1^ results from the stretching vibrations of the hydroxyl group (–OH) present in NHAP. Furthermore, a broad band that extends from 2500 to 3600 cm^− 1^ is associated with the stretching vibrations υ_3_ and υ_1_ of H_2_O molecules^34^. The peak observed at 1797 cm ^− 1^indicates the υ_2_ bending mode associated with H_2_O molecules. Furthermore, the absorption bands detected at 1422, 1593, and 872 cm^− 1^ in NHAP correspond to the vibrational modes of carbonate ions (CO_3_^2−^), which suggests the successful formation of a biological NHAP that resembles natural bone. Consequently, the FTIR spectrum of NHAP agrees with the literature, validating the effectiveness of the NHAP synthesis process^34,35^.

Figure 1a depicts the FT-IR spectra of ZnO NPs. Figure 1b illustrates the unique composition of NHAP.

#### X-ray diffraction (XRD)

**Fig. 2:**
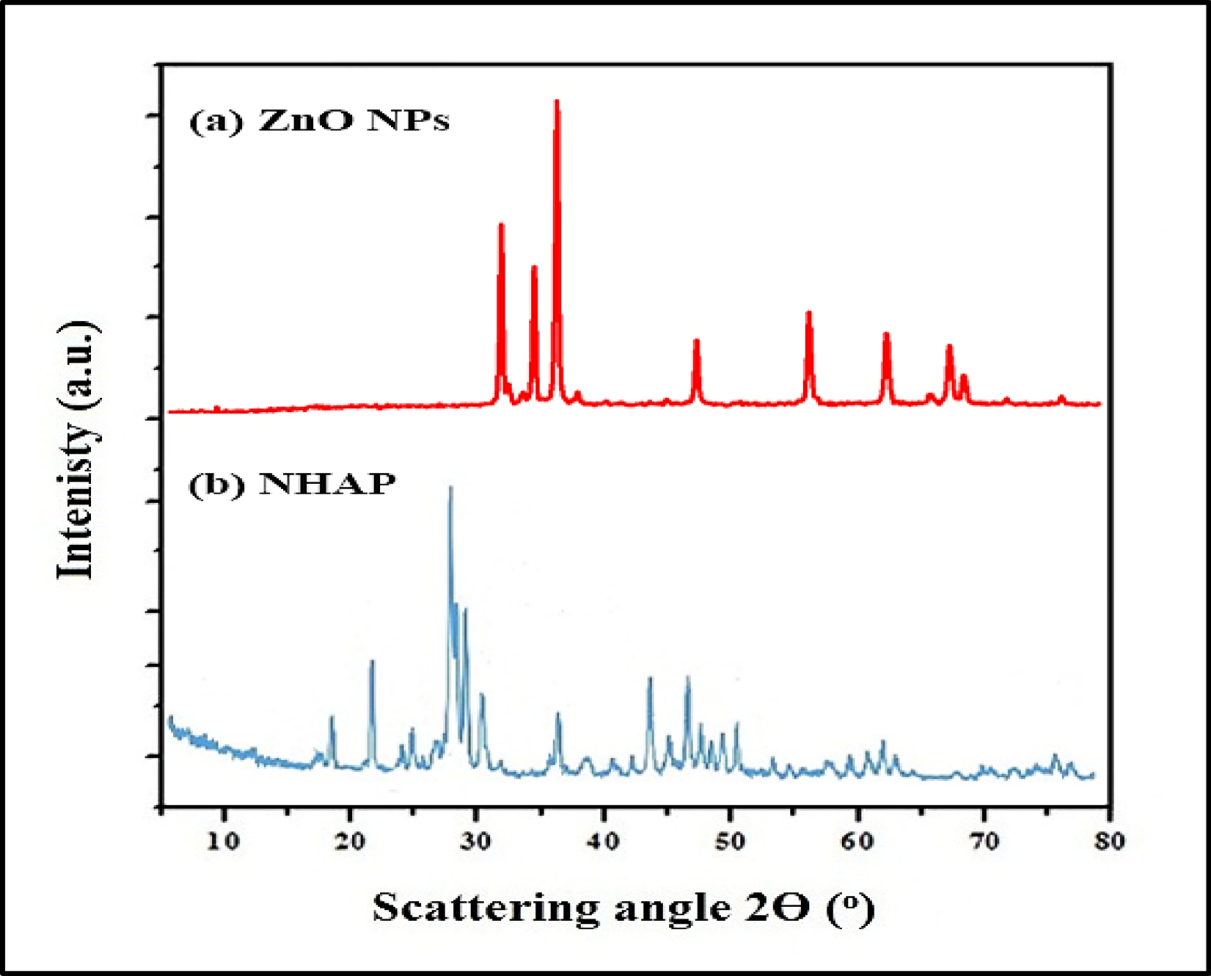
XRD patterns of (**a**) ZnO NPs exhibit peaks that align with Bragg reflections at 2θ values of 31.61°, 34.25°, 36.64°, 47.95°, 56.22°, 62.88°, 66.39°, 67.78°, and 68.99°. The corresponding Miller indices for these reflections are (100), (002), (101), (102), (110), (103), (200), (112), and (201). The observed diffraction pattern verifies the existence of ZnO in a hexagonal wurtzite structure, as referenced by the standard JCPDS card 36-1451^23^. The pronounced intensity and sharpness of these peaks reveal the crystalline characteristics of the prepared ZnO NPs. The absence of diffraction peaks related to impurities suggests that the synthesized product of high purity. (**b**) NHAP shows a reflection peak at 2θ values between 27.8° and 29.1°, consistent with the typical distinctive peak of the apatite phase (JCPDS card number 09–0432). This result signifies that the synthesis of NHAP powder has been successfully achieved. Furthermore, several notable peaks were identified at 2θ = 23°, which aligns with the (201) plane; at 2θ = 28.65°, relating to the (102) plane; at 2θ = 29.3°, associated with the (210) plane; and at 2θ = 35°, associated with the (301) plane of the NHAP sample. The formation of crystalline NHAP, which possesses a hexagonal phase structure, is supported by the Miller indices of the aircraft (102), (201), (210), and (301)^33^. These results imply that NHAP from eggshells consists of calcium carbonate with a distinctly defined and highly crystalline structure, showing no signs of secondary phase formation.

X-ray diffractograms for ZnO NPs are depicted in Fig. 2a, **and** the XRD diffractogram for NHAP in Fig. 2b.

#### Microstructural analysis using SEM and TEM

**Fig. 3:**
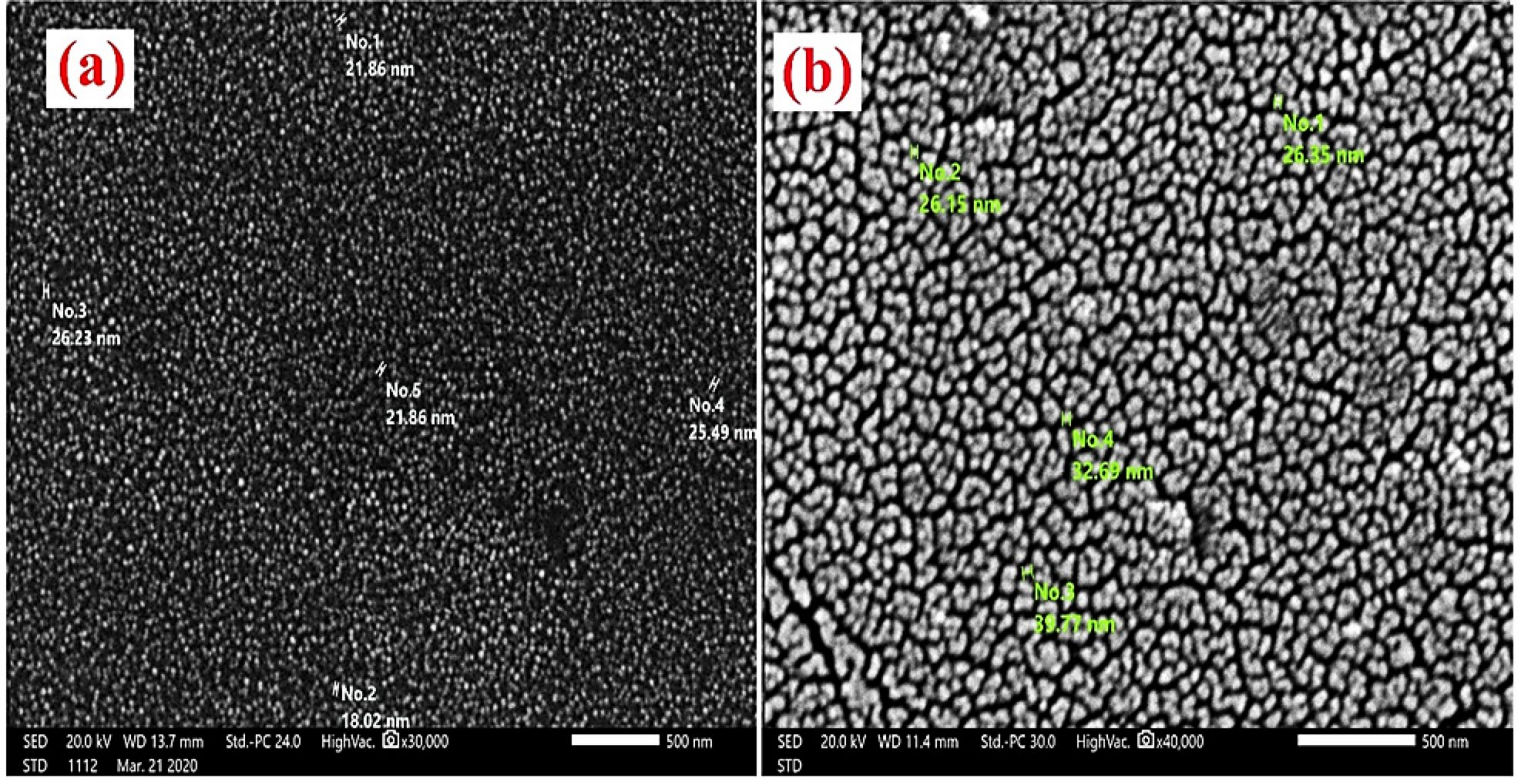
SEM images of (**a**) illustrate the synthesized ZnO NPs, which are characterized by their semi-spherical and hexagonal shapes. These primary particles tend to aggregate, leading to formations with an average size of 19.58 nm. (**b**) The NHAP particles resemble a hexagonal axial morphology, with particle sizes falling from 26.15 to 39.77 nm. The dimensions of the NHAP described above provide a substantial surface area, facilitating enhanced biological interactions with osteoblastic cells^36^. This form of NHAP is also regarded as a viable candidate for applications in bone tissue engineering scaffolds and a matrix for biosensors^37^.

**Fig. 4:**
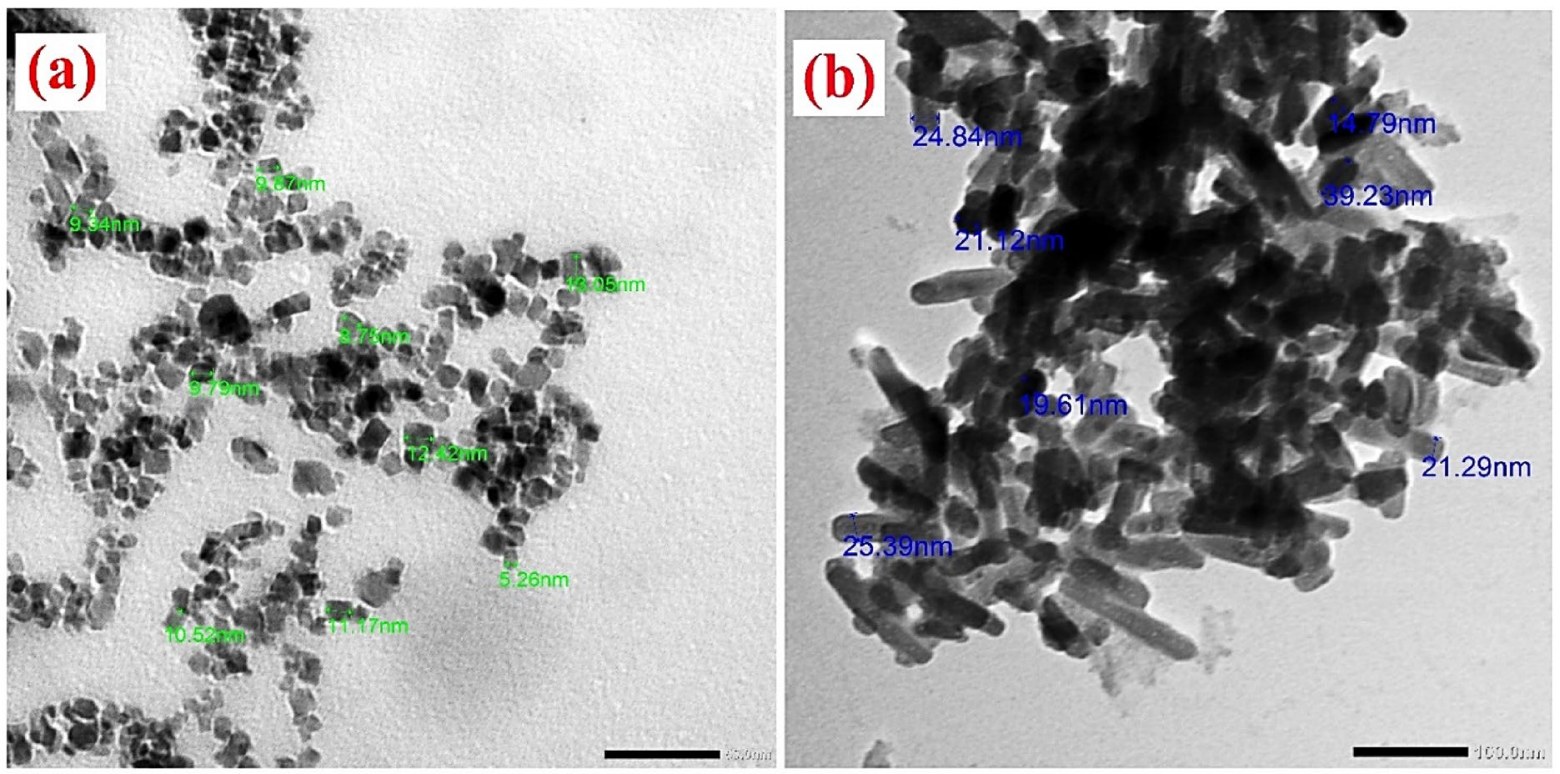
TEM images of (**a**) ZnO NPs displayes predominantly flaky hexagonal shapes, with an average size of 13.71 nm. The SEM image suggested that the agglomeration of the nanoparticles led to a size increase compared to the measurements obtained from TEM. This phenomenon primarily results from polarity and electrostatic interactions among the ZnO NPs^38^. The increase in size resulted from the particles overlapping with each other. This outcome agrees with the shape and uniformity characteristics of Zinc oxide nanoparticles^39^. (**b**) NHAP indicates that the sample analyzed showed a characteristic needle-like morphology, with particle dimensions between 14.97 and 39.23 nm. Moreover, the NHAP particles were observed to be non-agglomerated and uniformly dispersed.

Figure 3a and b display the SEM images of ZnO NPs and NHAP. The TEM images depicting ZnO NPs and NHAP are also shown in Fig. 4a and b. These images corroborate the findings from the SEM analysis, showing only a minor difference in particle size.

#### Energy dispersive X-rays (EDX)

**Fig. 5:**
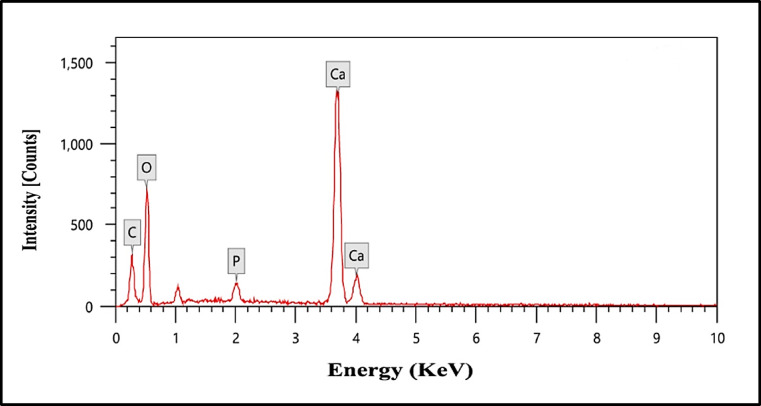
EDX spectrum of the prepared NHAP sample showing the Ca and P contents. The elemental analysis revealed that the sample tested had a Ca/P ratio of approximately 1.72, relatively near the standard value of 1.67, the theoretical ratio for apatite. This finding validates the practical synthesis of NHAP powder.

EDX analysis was conducted on the synthesized NHAP sample to verify its material composition before implantation (Fig. 5). The results displayed distinct peaks corresponding to oxygen (O), phosphorus (P), and calcium (Ca), along with minor traces of carbon (C) impurities. The carbonate was derived from the eggshells, which acted as the calcium source during the synthesis.

### Light microscopic results

**Fig. 6:**
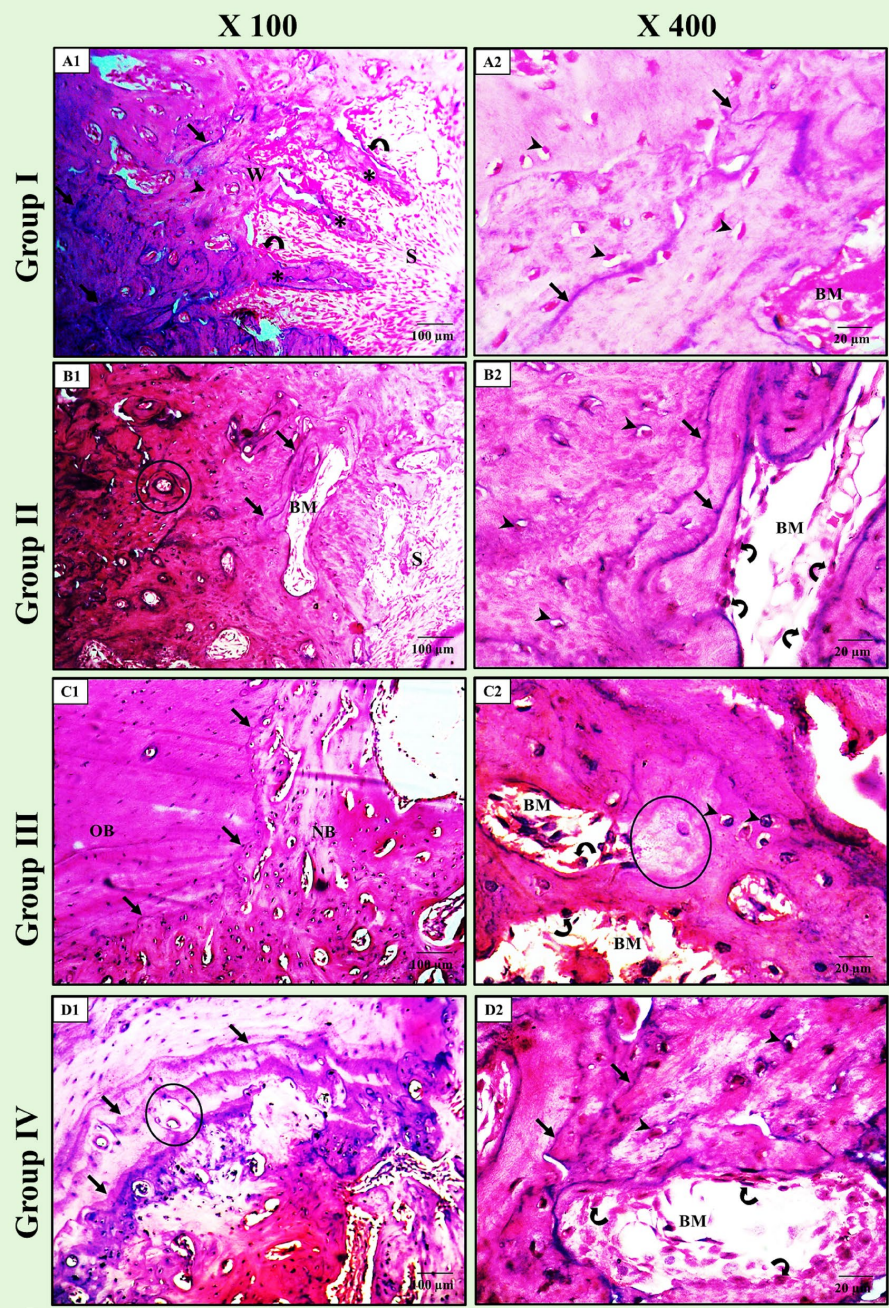
Light photomicrographs of the socket area for all groups. (**A1** and** A2**) showing the socket area of *group I (+ ve control)* in which the mature bone fills most of the socket area with bone trabeculae (*) radiating from the walls (W) of the socket (S), which appeared filled with fibrovascular granulation tissues. The trabeculae have active osteoblasts (curved arrows) on their surfaces. Also, multiple remodelling lines (arrows) and regularly distributed osteocytes in their lacunae (arrowheads) surrounding the vascular bone marrow (BM) can be depicted. (**B1** and** B2**) showing the socket area of *group II (ZnO NPs)*, in which the socket area (S) appeared filled with fibrous tissues. The osteons of the typical Haversian system (circled area) can be easily detected with obvious remodelling lines (arrows) surrounding well-vascularized BM spaces. Cellular activity can be described, including active plumb-shaped osteoblasts (curved arrows) lining the marrow spaces and numerous large osteocytes in their lacunae (arrowheads) with normal distribution. (**C1** and** C2**) showing the socket area of *group III (NHAP)*, in which the new bone trabeculae occupy a large area of the healing socket with large bone marrow spaces (BM) rich in blood supply. Note the complete fusion between the newly formed bone (NB) and old preexisting bone (OB) with an obvious line demarcating this fusion (black arrows). Active osteoblasts (curved arrows) line the BM spaces, and osteocytes with large nuclei occupy wide lacunae (arrow heads). Notice the newly formed bone area (circled area) undergoing mineralization. (**D1** and **D2**) showing the socket area of *group IV (ZnO NPs + NHAP)*, in which mature bone at the lateral wall of the healing socket with evident osteons (circled area) in the form of central Haversian canals surrounded by osteocytes, and numerous reversal lines (arrows) denoting areas of remodeling can be easily detected. While at the center of the socket, the trabecular bone shows large, highly vascularized bone marrow spaces (BM) lined with many osteoblasts (curved arrows). BM spaces are surrounded by osteocytes with large nuclei occupying wide lacunae (arrow heads). (H& E stain, A1, B1, C1 and D1 × 100; A2, B2, C2 and D2 × 400).

*In Group I (+ ve control)*, the mature bone appeared to fill most of the socket area with bone trabeculae radiating from the walls of the socket, which appeared filled with fibrovascular granulation tissues. The trabeculae showed a lining of active osteoblasts on their surfaces. Also, multiple resting/remodelling lines and regularly distributed osteocytes in their lacunae surrounding the vascular bone marrow were depicted (Fig. 6-A1 & A2).

The socket area in *Group II (ZnO NPs)* appeared filled with fibrous tissues. The osteons of the typical Haversian system with haversian canals were easily detected. Also, there are obvious resting/remodelling lines surrounding well-vascularized BM spaces. Cellular activity was described, including active plumb-shaped osteoblasts lining the marrow spaces and numerous large osteocytes in their lacunae with normal distribution (Fig. 6-B1 & B2).

In *Group III (NHAP)*, the new bone trabecula occupied a significant area of the healing socket with large bone marrow spaces and a rich blood supply. The complete fusion between the newly formed and old preexisting bone was prominent, separated by a line demarcating this fusion. Active osteoblasts line the marrow spaces, and osteocytes with large nuclei were depicted in broad lacunae. The newly formed bone area is undergoing mineralization and has appeared (Fig. 6-C1 & C2).

*In Group IV (ZnO NPs + NHAP)*, the mature bone at the lateral wall of the healing socket appeared with evident osteons in the form of central Haversian canals surrounded by osteocytes, and numerous reversal lines denoting remodeling areas were detected. While at the center of the socket, the trabecular bone showed large, highly vascularized bone marrow spaces lined with a significant number of osteoblasts. Osteocytes with large nuclei inside broad lacunae surrounded the marrow spaces (Fig. 6-D1 & D2).

### Masson’s trichrome stain results

**Fig. 7:**
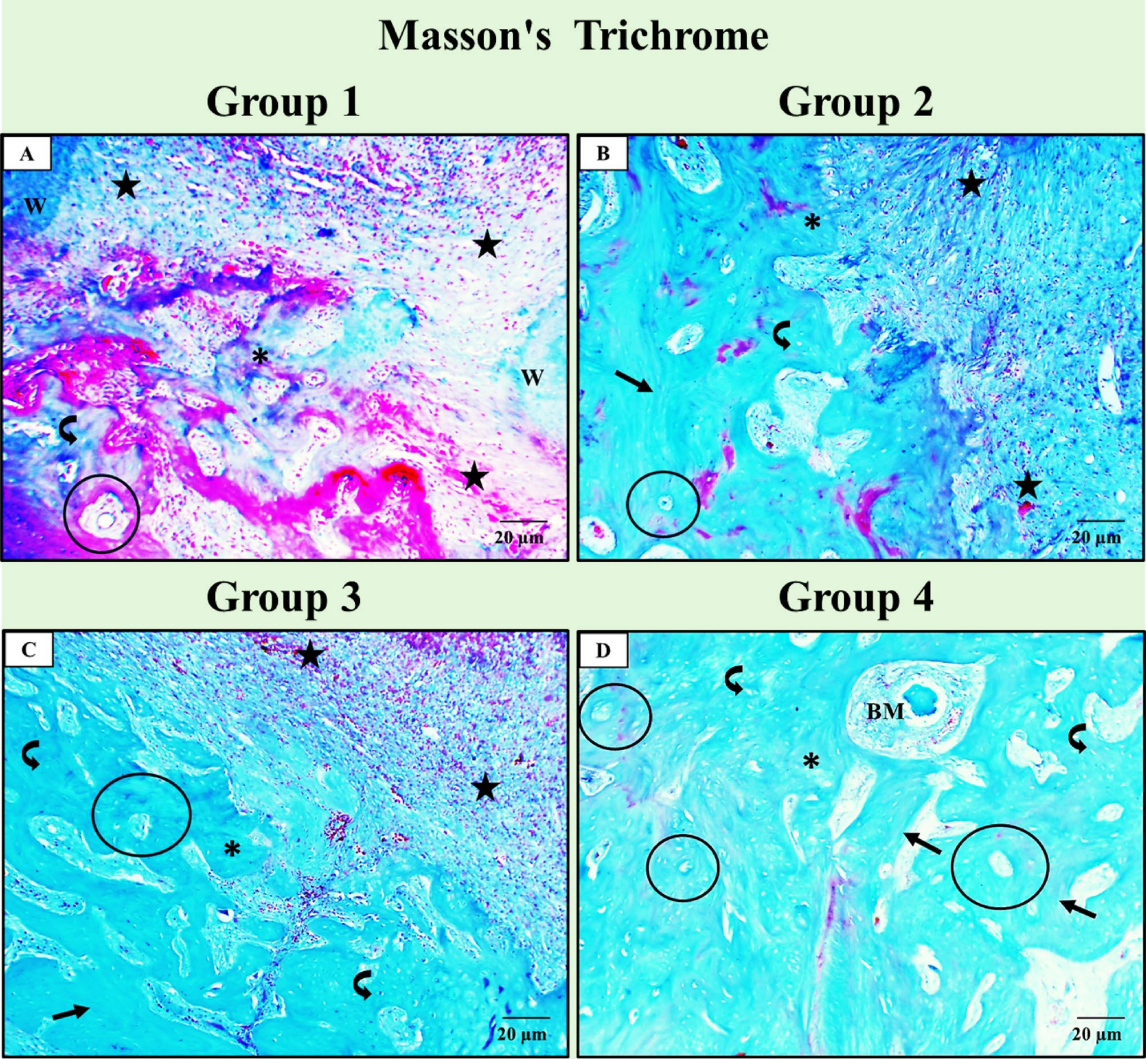
Light photomicrographs of Masson Trichrome stain for the socket area in all groups. (**A**) Group I (+ ve control) showing less bone formed inside the socket with thin bone trabeculae (*) and a large amount of fibrovascular granulation tissue (star) extending between the socket walls (W), fewer osteocytes (curved arrow), and no bone resting lines. (**B** and** C**) Group II (ZnO NPs) and Group III (NHAP). Both show thicker bone trabeculae (*), less granulation tissue (star), and a more obvious number of osteocytes (curved arrow) than in group I. Note the osteon formation inside the new bone (circled area). (**D**) Group IV (ZnO NPs + NHAP) depicts more bone formation with thicker bone trabeculae (*) around small bone marrow spaces (BM), more bone remodelling lines (arrow), more osteon formation (circled area), and more entrapped osteocytes (curved arrow) than the previous groups. (Masson Trichrome stain X 400).

*Group I (+ ve control)* showed the least bone formation inside the socket, with thin bone trabeculae and a large amount of fibrovascular granulation tissue extending between the socket walls. Also, fewer osteocytes were detected with no bone resting lines (Fig. 7-A). *Group II (ZnO NPs) and Group III (NHAP)* showed nearly equal results as they depicted thicker bone trabeculae, less granulation tissue, and a more obvious number of osteocytes than in Group I. The osteon formation inside the new bone was also detected (Fig. 7-B and C). However, *Group IV (ZnO NPs + NHAP)* depicted the highest results, as more bone formation with thicker bone trabeculae around small bone marrow spaces was detected easily. Also, bone resting/remodeling lines, osteon formation, and entrapped osteocytes appeared more than in the previous groups (Fig. 7-D).

### Scanning electron microscopic results

**Fig. 8:**
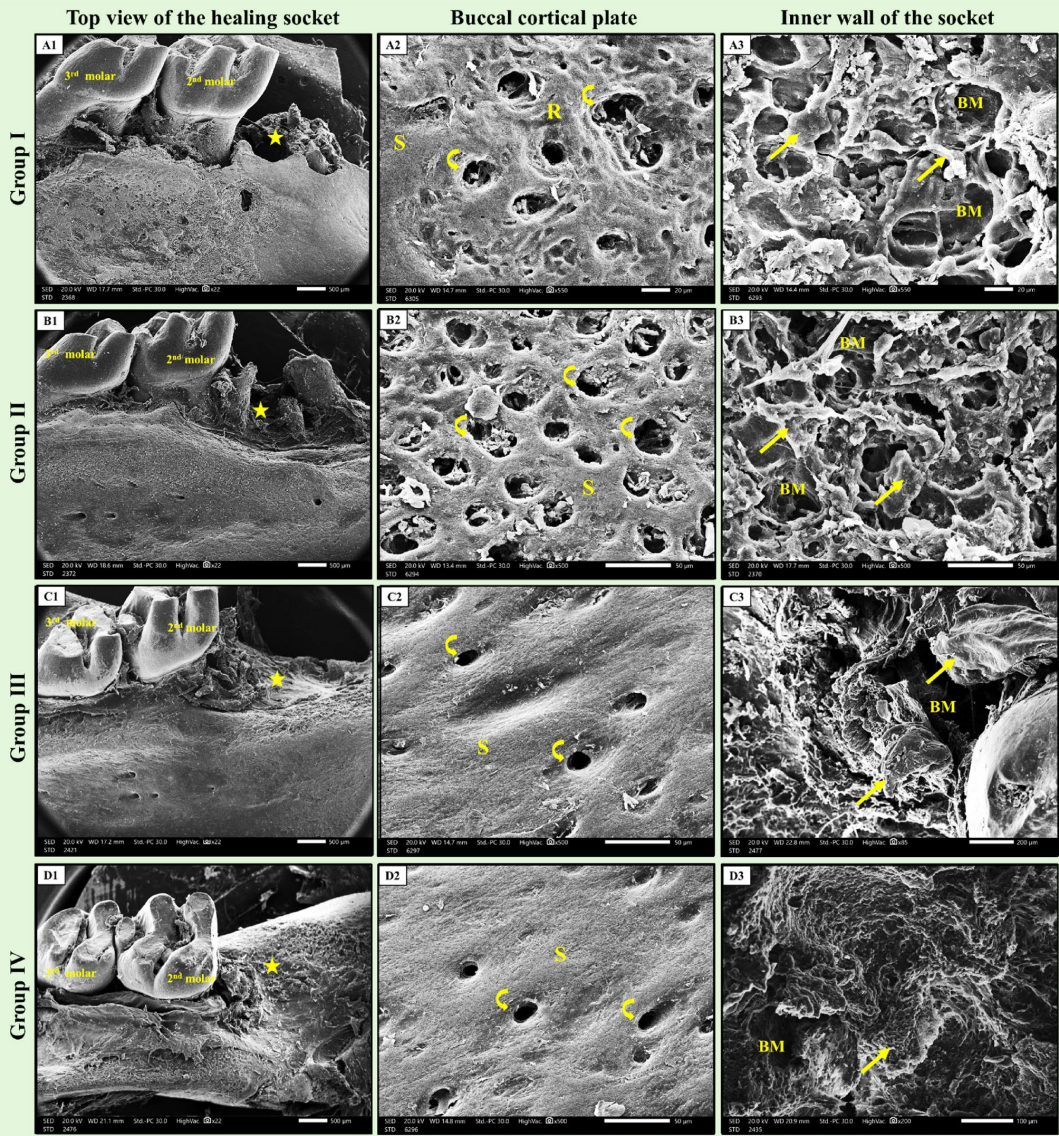
Scanning electron micrographs of the healing socket for all groups. (A1, B1, C1, and D1) showing top views of the healing socket. **A1**, *group I (+ ve control)* showing moderate healing with moderate socket closure (star) with bone formation. **B1**, *group II (ZnO NPs)*, shows a higher degree of healing with complete socket closure (star) and bone formation. **C1**, *group III (NHAP)*, showing complete healing with complete socket closure (star) and bone formation with a smooth surface. **D1**, *group IV (ZnO NPs + NHAP)* showing complete healing with complete socket closure (star) and bone formation with a smooth surface. (A2, B2, C2, and D2) showing higher magnification of the buccal cortical plate at the socket area. **A2**and **B2**, *group I and II*, respectively, show mixed smooth (S) and rough (R) surface topography with multiple nutritive canals (curved arrows), which show less regular surface outlines. **C2** and **D2**, *group III and IV*, respectively, show smooth (S) surface topography with numerous nutritive canals (curved arrows), which show more regular surface outlines. (A3, B3, C3, and D3) depicting the bone of the inner wall of the socket. **A3** and **B3**, *group I and II*, respectively, show newly formed bone consisting of layers of irregular thin trabeculae (arrows) separated by wide bone marrow spaces (BM). **C3** and **D3**, * group III and IV*, respectively, C3 shows that the freshly formed thick cortical bone trabeculae (arrows) surround smaller bone marrow spaces (BM). D3 depicts mature bone filling, but with areas of relatively thin and irregular trabeculae (arrows) in some fields. The bone appeared to fill the socket with obvious osteointegration with old bone. (SEM, original magnification; A1, B1, C1, and D1 × 500; A2, A3 × 20; B2, C2, D2, B3 × 50; C3 × 200; D3 × 100).

*Group I (+ ve control)* depicted moderate healing with moderate socket closure and bone formation. The buccal cortical plate of bone displayed mixed smooth and rough surface topography with multiple nutritive canals, which showed less regular surface outlines. The bone of the inner wall of the socket showed newly formed bone consisting of layers of irregular thin trabeculae separated by wide bone marrow spaces (Fig. 8-A1, A2 & A3).

*Group II (ZnO NPs)* showed a higher degree of healing with complete socket closure and bone formation. The buccal cortical plate of bone exhibited a mixed smooth and rough surface topography with multiple nutritive canals, which showed less regular surface outlines. The bone of the inner wall of the socket showed newly formed bone consisting of layers of irregular thin trabeculae separated by wide bone marrow spaces (Fig. 8-B1, B2 & B3).

*Group III (NHAP)* depicted complete healing, complete socket closure, and bone formation with a smooth surface. The buccal cortical plate of bone displayed smooth surface topography with numerous nutritive canals, which showed more regular surface outlines. The bone of the inner wall of the socket showed thick cortical bone trabeculae surrounding a smaller number of bone marrow spaces. The bone appeared to fill the socket with obvious osteointegration with the old bone (Fig. 8-C1, C2, & C3).

*Group IV (ZnO NPs + NHAP)* showed complete healing, complete socket closure, and bone formation with a smooth surface. The buccal cortical plate of bone displayed smooth surface topography with numerous nutritive canals, which showed more regular surface outlines. The bone of the inner wall of the socket showed mature bone filling, but with areas of relatively thin and irregular trabeculae in some fields. The bone appeared to fill the socket with obvious osteointegration with old bone (Fig. 8-D1, D2, & D3).

### Statistical results

#### Histomorphometric results of bone surface area

**Fig. 9:**
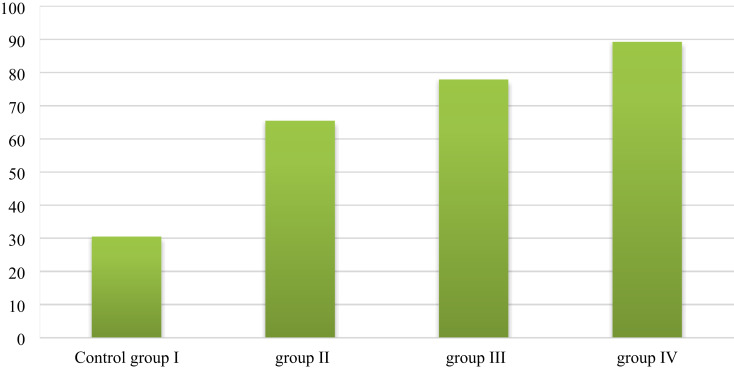
Bar chart for all groups showing the new bone surface area percentage 4 weeks after extraction.

Table 2; Fig. 9 show all groups’ new bone surface area percentages. The results are summarized as the means and standard deviations.

**Table 2:** Comparison of the percentage of new bone surface area after 4 weeks of extraction among the four studied groups.

Percentage of new bone	Group I (*n* = 3)	Group II (*n* = 3)	Group III (*n* = 3)	Group IV (*n* = 3)	F	*p*
Week 4						
Min.–Max.	30.30–34.70	65.20–69.30	71.10–79.60	86.20–90.50	135.704*	< 0.001*
Mean ± SD.	32.83 ± 3.50	67.07 ± 3.08	76.53 ± 5.82	88.33 ± 2.78		
Median	31.50	66.60	78.80	89.30		
p_**Control**_		< 0.001*	< 0.001*	< 0.001*		
Sig. bet. grps		p_1_ = 0.001^*^, p_2_ < 0.001^*^, p_3_ = 0.001^*^				

F: F for ANOVA test, pairwise comparison between. Two groups were analyzed using a post hoc test (Tukey).

After week 4 of tooth extraction, group IV (ZnO NPs and NHAP) had the highest percentage of new bone formation (88.33 ± 2.78), followed by group III (NHAP) (76.53 ± 5.82) and group II (ZnO NPs) (67.07 ± 3.08). In contrast, Group I (control) had the minimal percentage (32.83 ± 3.50). There was a statistically significant difference in the rate of newly formed bone between Group I (control) and the other three treatment groups, II, III, and IV (*p < 0.001*). Moreover, there was a statistically significant difference in the percentage of new bone among the three treatment groups (p1 < 0.001), (p2 < 0.001), and (p_3_ < 0.001), respectively.

#### Energy dispersive X-ray analysis (EDXA)of the bone socket results

**Fig. 10:**
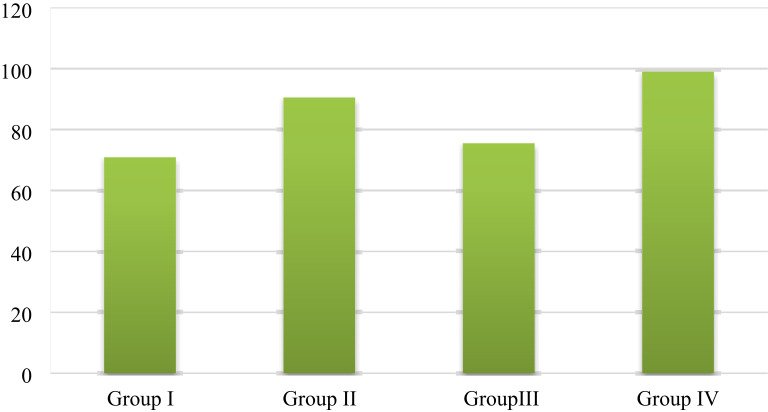
Bar chart for all groups showing the percentage of calcium within the socket bone at 4 weeks after tooth extraction.

**Fig. 11:**
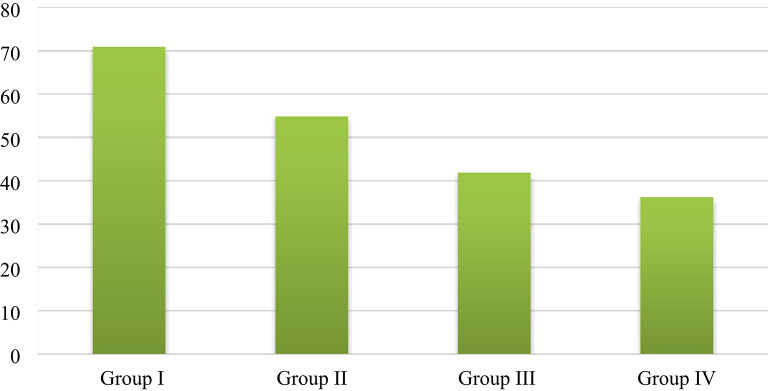
Bar chart for all groups showing the percentage of phosphorus within the socket bone at 4 weeks after tooth extraction.

After 4 weeks of extraction, the cells were examined by EDXA according to the percentages of Ca (Table 3; Fig. 10) and P (Table 4; Fig. 11). The results are summarized as the means and standard deviations as follows:

**Table 3:** Comparison of the percentage of calcium within the socket bone at 4 weeks after extraction between the four groups.

Percentage of calcium	Group I	Group II	Group III	Group IV	F	*p*
Week 4						
Min.–Max.	70.50–72.52	87.30–94.50	71.30–74.50	95.90–98.50	255.565*	< 0.001*
Mean ± SD.	70.70 ± 0.89	92.30 ± 2.69	76.16 ± 1.25	98.68 ± 2.20		
Median	70.90	90.50	75.50	99.0		
p_**Control**_		< 0.001*	0.627	< 0.001*		
Sig. bet. grps		p_1_ < 0.001*, p_2_ < 0.001*, p_3_ < 0.001*				

F: F for ANOVA test, Pairwise comparison bet. Two groups were analyzed using a post hoc test (Tukey).

**Table 4:** Comparison of the percentage of phosphorus within the socket bone at 4 weeks after extraction between the four groups.

Percentage of phosphorus	Group I	Group II	Group III	Group IV	F	*p*
Week 4						
Min.–Max.	60.50–61.90	42.10–44.60	53.0–57.10	35.30–45.60	492.012*	< 0.001*
Mean ± SD.	60.10 ± 0.50	41.90 ± 1.74	54.86 ± 2.15	36.22 ± 1.35		
Median	60.5	42.10	55.0	36.21		
p_**Control**_		< 0.001*	< 0.001*	< 0.001*		
Sig. bet. grps		p_1_ < 0.001*, p_2_ < 0.001*, p_3_ = 0.003*				

F: F for the ANOVA test. Pairwise comparisons between the groups were analyzed using a post hoc test (Tukey).

- Calcium percentage:
  Group IV (ZnO NPs and NHAP) displayed the uppermost Ca value (98.68 ± 2.20), followed by group II (ZnO NPs) (92.30 ± 2.69) and then group III (NHAP) (76.16 ± 1.25), while the control group showed the lowest Ca value (70.70 ± 0.89). There was a statistically significant decrease in calcium levels in the control group compared with group II (ZnO NPs) and group IV (ZnO NPs and NHAP) (p *<* 0.001). At the same time, there was no statistically significant difference between Group I (control) and Group III (NHAP) concerning calcium levels (p_Control_ = 0.627). In addition, there was a statistically significant difference in the Ca level between the other three treatment groups (p1 < 0.001), (p2 < 0.001), and (p_3_ < 0.001), respectively.
- Phosphorus percentage:
  Group I (control) had the highest p-value (60.10 ± 0.50), followed by group III (NHAP) (54.86 ± 2.15) and group II (ZnO NPs) (41.90 ± 1.74), while group IV (ZnO NPs and NHAP) had the lowest p-value (36.22 ± 1.35). There was a statistically significant increase in the p-value in the control group compared with the other groups (*p < 0.001)*. In addition, there was a statistically significant difference in the p-value between the other three treatment groups (p_1_ < 0.001), (p_2_ < 0.001), and (p_3_ < 0.001).

## Discussion

Worldwide, there is a trend towards a sustainable industry, and more efforts are being made to guarantee that the waste is recycled as much as possible. There is also an emphasis on adding value to new products. It has been shown that eggshells are considered a biocompatible material for medical applications. In addition, HAP can be synthesized from eggshell waste and used for bone regeneration applications^34,35^. Despite their advantages, HAP nanoparticles can be expensive to produce, so to advance nanotechnology without any economic strain, there is a clear need for large, inexpensive amounts of clinical-grade HAP^36^.

This study evaluated a simple chemical technique for synthesizing NHAP and its potential role in enhancing alveolar socket healing, separately and in combination with ZnO NPs. After reviewing many previous studies, this was the first attempt to assess the effect of combining ZnO NPs and NHAP.

As evidence of the method’s success in NHAP preparation, the phase purity and presence of major functional groups in the HAP powder were tested by FTIR analyses, which confirmed the presence of the pure apatite phase. Carbonate (CO_3_^2−^) also appeared in the sample, detected at 1422, 1593, and 872 cm^− 1^ absorption bands. The presence of CO_3_^2−^ ions in biological apatite is significant because it is the primary source of lattice distortion, creating micro stresses and crystalline defects in the surrounding area, which, in turn, play a vital role in its solubility, which is considered favorable for bone remodeling. Thus, synthetic apatite should exhibit a small particle size and CO_3_^2−^ to produce superior bone formation and maturation properties^37^. It is also quite interesting to note that all the samples investigated by TEM and XRD confirmed the nano-dimensions of the synthesized NHAP, like the shape of bone-derived HAP (∼20 nm in width)^38^.

The study results indicate that the NHAP/ZnO NPs mixture is suitable for potential usage as a bone replacement material in quick-hardening paste. Although the exact synergistic effect is not entirely understood, the interaction between NHAP and ZnO NPs might be explained by the fact that the zinc oxide nanoparticles influence the properties of the obtained material by promoting bone growth, inhibiting bone resorption, and acting as antibacterial agents inside the alveolar socket. Furthermore, studies of the interaction between Zn and NHAP showed that an apatite layer developed on the material’s surface when Zn-HAP was submerged in simulated bodily fluid (SBF), and the layer’s thickness rose as the Zn level rose, suggesting that Zn promoted mineralisation and may enhance HA’s bioactivity. This paste may lessen soft tissue irritation when used clinically as a bone replacement in dental treatment^39^.

Among the experimental animals in this study, males were selected due to their small size, availability, and timing of the bone remodeling cycle, which seems like that of humans. This finding was in line with a previous study^40^. Still, it was not in agreement with Covani et al.^41^, who reported that using rabbits might be much better as a model for extraction because rabbits are larger and have a rapid healing cycle. The first mandibular molar was chosen as the tooth model in the current study because rodent incisors continuously erupt, which might change the anatomy of the alveolar socket^42^. The graft material in our research was applied directly inside the socket. The application method was chosen according to Gharib et al ^43^. The time point in the current study was selected to be 4 weeks after tooth extraction, which was a suitable time of analysis of the final stages of healing. Also, it was chosen because, in rat alveolar socket models, bone maturation and marked mineralization suitable for histomorphometric and EDX evaluation are typically evident at 4 weeks. However, earlier points (e.g., 2 weeks) represent predominantly early granulation and woven bone stages^44^.

Histological examination of bone tissue was performed using H&E staining, followed by histomorphometric analysis to confirm the histological findings during bone tissue examination^45^. According to Ellingham et al. ^46^, SEM/EDX is a rapid diagnostic tool for identifying bone surfaces and components. Thus, in the present study, SEM was used to examine the surface of the new bone that formed inside the healing socket, while EDXA was used for elemental analysis.

As a central part of many related studies, the + ve control group was used as a baseline for comparison with other test groups. The control group’s results in the present study agree with those of Avila-Ortiz et al.^47^, who reported that untreated extraction sites often have poorer mineralization and bone quality than other test groups.

SEM, EDXA, and histomorphometric analysis confirmed the results of the histological examination of the ZnO NPs group. The healing socket in this Group showed dense, mature bone filling the larger part of the socket. This can be explained by the findings of many recent studies suggesting that ZnO NPs potentiate bone healing. ZnO NPs can activate all signaling cascades related to mesenchymal cell commitment, osteoblast differentiation, and proliferation, and directly promote osteogenesis through actions on the Runt-related transcription factor 2 (RUNX2) gene^48^.

Regarding the results of the NHAP group, the findings showed that the healing of the extraction socket progressed faster than that of the control and ZnO NPs groups, with a greater percentage of new bone formation and a remarkable increase in bone vascularity. These findings agree with the observations of Abniki^49^ and Hassan and El-Sayed El-Haddad^50^. Also, Helal et al.^51^ concluded that eggshell powder had a higher remineralization potential when used in enamel remineralization after artificially induced enamel carious lesions.

An examination of group IV (ZnO NPs + NHAP groups) in the present study showed the best bone quality and healing duration results among the study groups. Several mechanisms have been proposed to explain the outstanding results of the interaction between ZnO NPs and NHAP, including increased production of growth factors and expression of transcription factors that regulate osteoblast differentiation^52^. These findings agree with those of Bahammam et al.^53^ and Hasri et al.^54^, who suggested that ZnO NPs and NHAP could enhance the early release of growth factors that increase angiogenesis and regeneration in healing sockets. Additionally, these findings may be attributed to the anti-inflammatory effect of the ZnO NPs through the activation of enzymatic antioxidants and the removal of free radicals. These results agree with those of Hu et al^55^.

## Conclusions and recommendations

Treatment modalities with either ZnO NPs or NHAP administration could improve bone socket healing. However, combining ZnO NPs and NHAP resulted in better histological and statistical results. This might be due to the enhancement of the early release of growth factors that increase angiogenesis and regeneration in healing sockets. However, additional studies are needed to evaluate this combination. Moreover, to build upon these findings, we suggest that future investigations utilize organ-on-a-chip (OOAC) platforms to clarify the underlying cellular and molecular mechanisms driving the enhanced bone healing response in ZnO NPs and NHAP mixture.

## **Study limitations**

 While this study focused on histology, histomorphometry, SEM, and EDX as the initial characterization analysis of bone healing, the absence of immunohistochemical and molecular markers (e.g., osteocalcin, bone alkaline phosphatase, and Runx2) is a limitation. Future studies, including immunohistochemical and molecular assays, will further clarify how NHAP and ZnO NPs affect osteogenesis. Future studies with multiple time points (e.g., 2 and 6 weeks) should profile temporal changes in more detail.

## Data Availability

All data generated or analyzed during this study are included in the submitted files.
